# *TYK2* Protein-Coding Variants Protect against Rheumatoid Arthritis and Autoimmunity, with No Evidence of Major Pleiotropic Effects on Non-Autoimmune Complex Traits

**DOI:** 10.1371/journal.pone.0122271

**Published:** 2015-04-07

**Authors:** Dorothée Diogo, Lisa Bastarache, Katherine P. Liao, Robert R. Graham, Robert S. Fulton, Jeffrey D. Greenberg, Steve Eyre, John Bowes, Jing Cui, Annette Lee, Dimitrios A. Pappas, Joel M. Kremer, Anne Barton, Marieke J. H. Coenen, Barbara Franke, Lambertus A. Kiemeney, Xavier Mariette, Corrine Richard-Miceli, Helena Canhão, João E. Fonseca, Niek de Vries, Paul P. Tak, J. Bart A. Crusius, Michael T. Nurmohamed, Fina Kurreeman, Ted R. Mikuls, Yukinori Okada, Eli A. Stahl, David E. Larson, Tracie L. Deluca, Michelle O'Laughlin, Catrina C. Fronick, Lucinda L. Fulton, Roman Kosoy, Michael Ransom, Tushar R. Bhangale, Ward Ortmann, Andrew Cagan, Vivian Gainer, Elizabeth W. Karlson, Isaac Kohane, Shawn N. Murphy, Javier Martin, Alexandra Zhernakova, Lars Klareskog, Leonid Padyukov, Jane Worthington, Elaine R. Mardis, Michael F. Seldin, Peter K. Gregersen, Timothy Behrens, Soumya Raychaudhuri, Joshua C. Denny, Robert M. Plenge

**Affiliations:** 1 Division of Rheumatology, Immunology, and Allergy, Brigham and Women's Hospital, Harvard Medical School, Boston, Massachusetts, United States of America; 2 Division of Genetics, Brigham and Women's Hospital, Harvard Medical School, Boston, Massachusetts, United States of America; 3 Program in Medical and Population Genetics, Broad Institute, Cambridge, Massachusetts, United States of America; 4 Partners HealthCare Center for Personalized Genetic Medicine, Boston, Massachusetts, United States of America; 5 Department of Biomedical Informatics, Vanderbilt University, Nashville, Tennessee, United States of America; 6 ITGR Human Genetics Group, Genentech Inc, San Francisco, California, United States of America; 7 The Genome Institute, Washington University School of Medicine, St. Louis, Missouri, United States of America; 8 New York University Hospital for Joint Diseases, New York, New York, United States of America; 9 Arthritis Research UK Epidemiology Unit, University of Manchester, Manchester Academic Health Sciences Centre, Manchester, United Kingdom; 10 The Feinstein Institute for Medical Research, North Shore-Long Island Jewish Health System, Manhasset, New York, United States of America; 11 Columbia University, College of Physicians and Surgeons, New York, New York, United States of America; 12 The Albany Medical College and The Center for Rheumatology, Albany, New York, United States of America; 13 Radboud university medical center, Radboud Institute for Health Sciences, Department of Human Genetics, Nijmegen, The Netherlands; 14 Radboud University Medical Center, Donders Centre for Neurosciences, Department of Psychiatry and Human Genetics, Nijmegen, The Netherlands; 15 Radboud University Medical Center, Radboud Institute for Health Sciences, Nijmegen, The Netherlands; 16 Université Paris-Sud, Orsay, France; 17 APHP–Hôpital Bicêtre, INSERM U1012, Le Kremlin Bicêtre, Paris, France; 18 Rheumatology Research Unit, Instituto de Medicina Molecular, Faculdade de Medicina da Universidade de Lisboa, Lisbon, Portugal; 19 Rheumatology Department, Santa Maria Hospital–CHLN, Lisbon, Portugal; 20 Amsterdam Rheumatology and Immunology Center, Department of Clinical Immunology & Rheumatology, Academic Medical Center /University of Amsterdam, Amsterdam, The Netherlands; 21 Laboratory of Immunogenetics, Department of Medical Microbiology and Infection Control, VU University Medical Center, Amsterdam, The Netherlands; 22 Amsterdam Rheumatology and Immunology Center, Department of Rheumatology, Reade, Amsterdam, The Netherlands; 23 Department of Rheumatology, Leiden University Medical Centre, Leiden, The Netherlands; 24 Division of Rheumatology and Immunology, Omaha VA and University of Nebraska Medical Center, Omaha, Nebraska, United States of America; 25 Department of Biochemistry and Molecular Medicine, University of California Davis, Davis, California, United States of America; 26 Information Systems, Partners Healthcare, Charlestown, Massachusetts, United States of America; 27 Instituto de Parasitologia y Biomedicina Lopez-Neyra, CSIC, Granada, 18100, Spain; 28 Genetics Department, University Medical Center and Groningen University, Groningen, The Netherlands; 29 Rheumatology Unit, Department of Medicine, Karolinska Institutet and Karolinska University Hospital Solna, Stockholm, Sweden; 30 Department of Medicine, Vanderbilt University, Nashville, Tennessee, United States of America; National Institute of Dental and Craniofacial Research, UNITED STATES

## Abstract

Despite the success of genome-wide association studies (GWAS) in detecting a large number of loci for complex phenotypes such as rheumatoid arthritis (RA) susceptibility, the lack of information on the causal genes leaves important challenges to interpret GWAS results in the context of the disease biology. Here, we genetically fine-map the RA risk locus at 19p13 to define causal variants, and explore the pleiotropic effects of these same variants in other complex traits. First, we combined Immunochip dense genotyping (n = 23,092 case/control samples), Exomechip genotyping (n = 18,409 case/control samples) and targeted exon-sequencing (n = 2,236 case/controls samples) to demonstrate that three protein-coding variants in *TYK2 (tyrosine kinase 2)* independently protect against RA: P1104A (rs34536443, OR = 0.66, P = 2.3x10^-21^), A928V (rs35018800, OR = 0.53, P = 1.2x10^-9^), and I684S (rs12720356, OR = 0.86, P = 4.6x10^-7^). Second, we show that the same three *TYK2* variants protect against systemic lupus erythematosus (SLE, P_omnibus_ = 6x10^-18^), and provide suggestive evidence that two of the *TYK2* variants (P1104A and A928V) may also protect against inflammatory bowel disease (IBD; P_omnibus_ = 0.005). Finally, in a phenome-wide association study (PheWAS) assessing >500 phenotypes using electronic medical records (EMR) in >29,000 subjects, we found no convincing evidence for association of P1104A and A928V with complex phenotypes other than autoimmune diseases such as RA, SLE and IBD. Together, our results demonstrate the role of TYK2 in the pathogenesis of RA, SLE and IBD, and provide supporting evidence for TYK2 as a promising drug target for the treatment of autoimmune diseases.

## Introduction

Human genetics has the potential to identify biological pathways that lead to complex diseases such as rheumatoid arthritis (RA). Meta-analyses of multi-ethnic genome-wide association studies (GWAS) in RA have now identified more than 100 loci associated to risk of disease [1]. Despite the success of GWAS, the associated loci usually include several genes in the region of linkage disequilibrium (LD), thus providing limited information to incriminate the causal genes. Cis-eQTL effects of SNPs that are in LD with the index SNPs have been reported in immune cell types, often describing association with variation of expression of several genes in the locus. Additionally, only a few RA loci harbour missense variants [1,2]. Even then, however, it is not clear if these SNPs are responsible for the signal of association, thus illustrating the challenges of interpreting GWAS findings to provide insights into the disease biology [3,4].

Several studies have described genes in GWAS loci that harbour multiple independent functional mutations associated with a disease, providing genetic evidence for causality and, by extension, insight into disease pathogenesis [5–18]. This highlights the critical need for detailed analyses combining dense genotyping and sequencing to pinpoint causal genes with an allelic series of associated protein-coding functional variants. In addition, this approach has the potential to clarify disease mechanisms and to identify novel therapeutic targets to guide drug discovery [19,20].

Allelic pleiotropy, where one genetic variant influences several distinct phenotypes, is increasingly recognized as a common phenomenon from GWAS findings [21], especially in the field of immune-mediated diseases [22]. Investigation of pleiotropic effects can inform disease biology and predict potential adverse events of targets derived from human genetics [20–22]. One approach to comprehensively investigate pleiotropy is through genotype data linked to clinical data derived from electronic medical records (EMR) [23]. This unbiased approach, called phenome-wide association study (PheWAS), allows for genotypes of interest to be tested for association to hundreds of clinically-relevant phenotypes [24]. We and others have demonstrated the value of this approach in successfully replicating results from GWAS and assessing pleiotropic effects [25–29].

One locus that has emerged from GWAS in RA is on chromosome 19p13 [2]. This locus spans 286 kb (LD region r^2^>0.5) and contains 11 genes, including *TYK2 (tyrosine kinase 2)*, a member of the Janus kinase (JAK) family of proteins that mediates signalling downstream of several cytokine receptors [30,31], and intercellular adhesion molecule (ICAM)–coding genes (*ICAM1*, *ICAM3*, *ICAM4*, *ICAM5*), which are part of the immunoglobulin superfamily. Based on the biology of RA alone [32], *TYK2* and *ICAM* genes are equally likely candidate genes responsible for the signal of association. In support of *TYK2*, the signal of association is driven by a low-frequency missense variant in *TYK2*, with a reported odds ratio (OR) of 0.62, protective for RA [2]. The variant, rs34536443 (p.P1104A), is predicted to be damaging using function prediction tools, and has been reported to be loss-of-function (LOF), affecting TYK2 kinase activity in primary T cells, fibroblast cell lines and B cell lines [33,34].

Genetic variation in the 19p32/*TYK2-ICAM* locus has also been associated with several other autoimmune diseases, including psoriasis, multiple sclerosis (MS), Type 1 diabetes (T1D), Crohn’s disease (CD), ulcerative colitis, and systemic lupus erythematosus (SLE) [35–42]. However, the leading signals of association differ in terms of implicated SNPs, effect sizes and directions of effect (protection vs risk). Whether these differences in association signals in autoimmune diseases refer to distinct causal variants and/or causal genes, or pleiotropic effects of the same variants remains unclear.

In the current study, we performed a detailed analysis of the 19p32/*TYK2-ICAM* locus to comprehensively investigate the contribution of common and rare protein-coding variants to RA susceptibility using 1) dense genotyping of the locus with the Immunochip and Exomechip genotyping platforms and 2) exon sequencing of all 11 genes within this locus. We then used Exomechip data to investigate the association of *TYK2* missense variants with two additional autoimmune diseases, SLE and IBD, both of which have been reported previously to harbour associations to genetic variants in this locus [35–38,40]. Finally, we linked our findings with electronic medical records (EMR) to comprehensively assess pleiotropic effects of the RA-associated *TYK2* missense variants.

## Results

### Three independent *TYK2* protein-coding variants protect against RA

**Fig 1:**
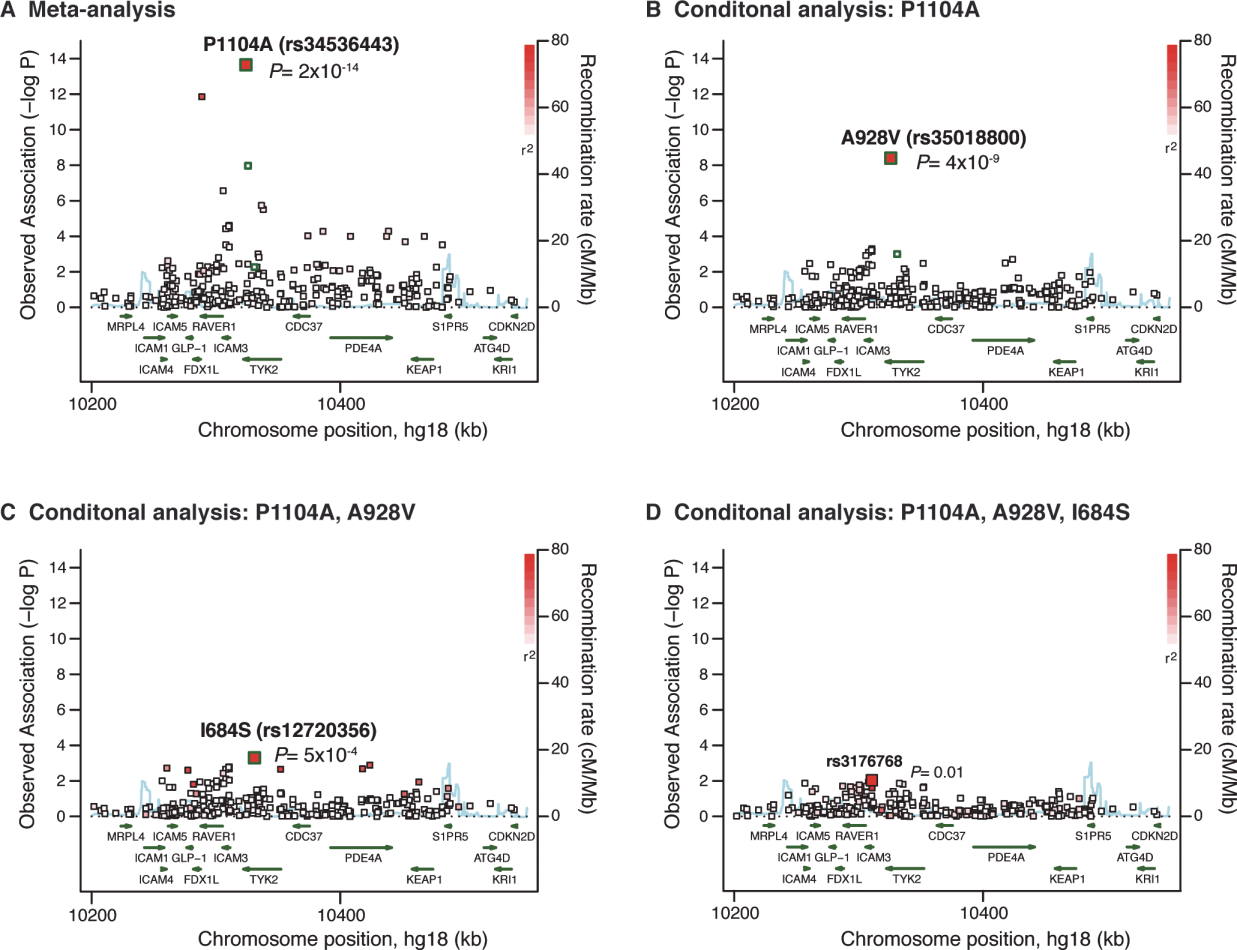
Results from stepwise conditional analysis of the *TYK2* locus. We fine-mapped the *TYK2* locus using Immunochip data available for 7,222 ACPA+ RA cases and 15,870 controls (MAF>0). (A) In the meta-analysis, the best signal of association was at the *TYK2* missense variant P1104A (rs34536443).(B) Conditional on P1104A, the best signal of association was at the *TYK2* missense variant A928V (rs35018800). (C) Conditional on P1104A and A928V variants, the best signal of association is at the *TYK2* missense variant I684S (rs12720356). (D) Conditional on the 3 RA-protective variants in *TYK2*, we observed no additional signal of association at the locus (best signal is rs3176768, P = 0.01). P-values from meta-analyses of logistic regressions results from 6 Immunochip collections are shown. The three *TYK2* missense variants predicted to be damaging and independently associated with RA risk are highlighted in green.

**Fig 2:**
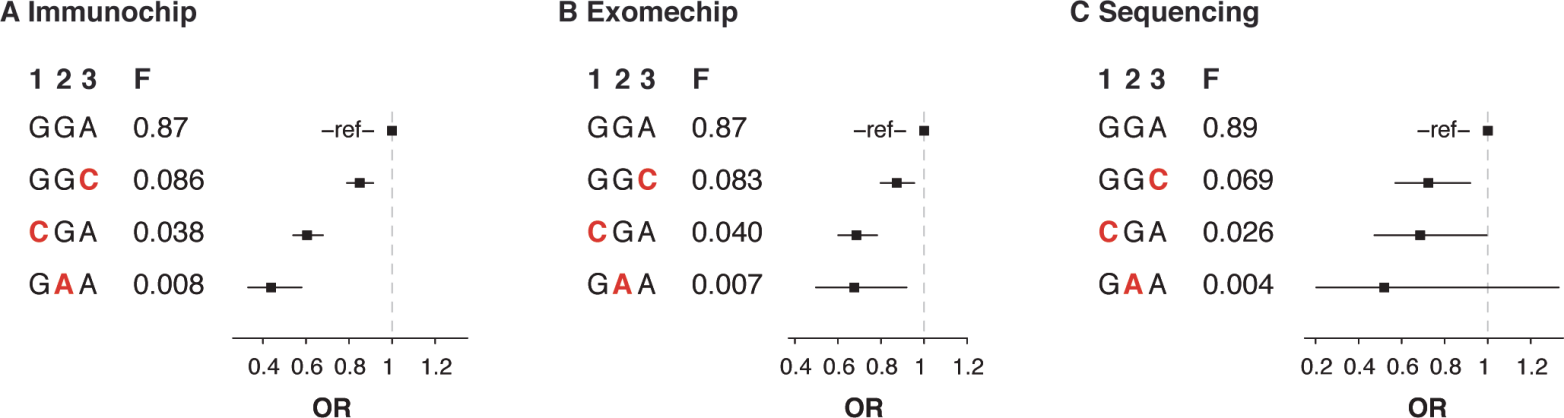
Contribution of 3 independent *TYK2* protein-coding variants to protection from RA. (A) Three variants with MAF>0.5% predicted to be damaging and protecting from RA (P1104A, A928V and I684S) were identified using Immunochip data for 7,222 ACPA+ RA cases and 15,870 controls of European ancestry. (B) The three variants were genotyped in an independent dataset on the Exomechip (4,726 RA cases, 13,683 controls). (C) The three variants genotypes were also available from exon sequencing of *TYK2* in 1,118 RA cases, 1,118 matched controls of European ancestry. Frequencies of the independent haplotypes and odds ratios (OR) relative to the most frequent haplotype are shown. Minor alleles of the variants are highlighted in red. H, haplotypes; F, haplotype frequency; 1, P1104A; 2, A928V; 3, I684S.

To fine-map the 19p32/*TYK2-ICAM* locus, we first performed a stepwise conditional analysis using Immunochip genotype data available for 7,222 ACPA+ RA cases and 15,870 controls of European ancestry (S1 Table) [2]. In this analysis, we applied no minor allele frequency (MAF) cut-off, in order to investigate all variants at the locus. The strongest signal was at the previously reported *TYK2* missense variant rs34536443 (P1104A, minor allele frequency [MAF] = 3.4%, OR = 0.62, P = 2.2x10^-14^) (Fig 1A and S2 Table) [2]. After conditioning on the P1104A variant, we observed a significant association at P = 4.0x10^-9^ with an OR = 0.42, driven by the rare *TYK2* missense variant A928V (rs35018800, MAF = 0.8%) (Fig 1B and S2 Table). Conditional on both *TYK2* P1104A and A928V variants, we observed a third signal of association at P = 5.4x10^-4^ (OR = 0.87), driven by the *TYK2* missense variant I684S (rs12720356; MAF = 8%) (Fig 1C and S2 Table) [2]. After conditioning on *TYK2* P1104A, A928V and I684S variants, we observed no association at P<0.01 (Fig 1D). We used the genotypes from the three *TYK2* variants P1104A, A928V and I684S to build haplotypes. The haplotype model confirmed independence of the variants, with the minor alleles of the three variants lying on different haplotypes (Fig 2A). All three missense variants were predicted to be damaging using Polyphen-2 and SIFT [43,44].

To replicate the association signals at the three *TYK2* missense variants, we used Exomechip genotype data in an independent set of 4,726 RA cases and 13,683 controls of European ancestry. We also directly sequenced the coding exons of *TYK2* in an independent set of 1,118 RA cases and 1,118 matched controls of European ancestry (S1 Table). In both the Exomechip and the sequencing data, the effect sizes of the protective haplotypes built using the P1104A, A928V and I684S variants were highly similar to the effect sizes observed in the Immunochip data, and were significantly associated with protection from RA (P_omnibus_ = 4.6x10^-8^ in Exomechip; P_omnibus_ = 0.0058 in sequencing) (Fig 2B and 2C). Meta-analysis of the Immunochip, Exomechip and sequencing datasets confirmed replication of the three independent association signals in *TYK2* (S2 Table). While both the P1104A and A928V variants reached genome-wide significance (P_META_ = 2.3x10^-21^ and P_META_ = 1.2x10^-9^, respectively), we estimated that a sample size of >20,000 RA cases would be required to observe an association at the I684S variant at genome-wide significance (P<5x10^-8^), based on the frequency and estimated effect size of the variant.

Together, these data implicate *TYK2* rather than one of the *ICAM* (or other) genes in the region of LD as the most likely causal gene responsible for the signal of association.

### Contribution of rare *TYK2* protein-coding variants to RA

To comprehensively investigate the contribution of rare protein-coding variants, we analysed exon-sequencing data available for the 11 genes in the 19p32/*TYK2-ICAM* locus in 1,118 RA cases and 1,118 matched controls of European ancestry (S1 and S3 Tables). We restricted the analysis to protein-coding variants (nonsense or missense) with MAF<0.5%, thus excluding the *TYK2* P1104A, A928Vand I684S variants.

**Fig 3:**
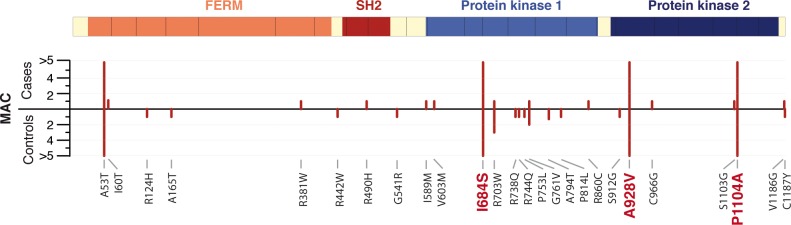
*TYK2* protein-coding variants identified by exon-sequencing of RA cases and controls. Using dense genotyping, we demonstrate that three *TYK2* protein-coding variants predicted to be damaging, P1104A, A928V, and I684S, protect against RA (highlighted in red). By exon-sequencing in 1,118 RA cases and 1,118 controls, we identified 23 additional missense variants predicted to be damaging (PolyPhen-2 and SIFT), with no strong evidence of association to RA in gene-based association tests. The *TYK2* coding exons, the protein domains, and the minor allele count (MAC) of the rare variants (MAC<5) in cases and controls are shown.

We first performed gene-based association tests for each of the 11 genes (assessing significance using 10,000 permutations of case-control status), using 4 different methods: the burden test (BURDEN, one-sided test), the frequency weighted test (WT, one-sided test), the variable threshold test (VT, one-sided test), and SKAT-O (two-sided test) [45–47]. Using the one-sided methods, we performed two tests, to assess the accumulation of rare variants in cases and controls, respectively. We found no gene harbouring rare variants significantly associated with RA (at P<0.004, 0.05/11 genes), in either gene-based tests including all protein-coding variants or restricted to variants predicted to be damaging (P>0.01; Fig 3 and S4 Table).

We then performed a sliding-window test. For each position in the protein-coding sequence of the 11 genes, we extracted all rare missense and nonsense variants in a 500 bp window centered on the position, and performed a window-based association test using SKAT-O (assessing significance using 1,000 permutations of case-control status; S1 Fig). We only observed a suggestive association at *TYK2* resulting from an accumulation of rare missense variants predicted to be damaging (7 variants, including 5 singletons) in controls in the protein kinase 1 domain–coding region (P = 0.016) (Fig 3, S1 Fig, S4 and S5 Tables).

We also investigated the contribution of all *TYK2* protein-coding variants genotyped in the Exomechip in our collection of 4,726 RA cases and 13,683 controls. We observed no additional single variant associated to RA beyond P1104A, A928Vand I684S (P>0.05; S2 Fig), and no aggregate signal of association driven by *TYK2* variants with MAF<0.5% using SKAT-O (P = 0.80).

Together, these results support the finding that the *TYK2* protein-coding variants P1104A, A928V and I684S variants are responsible for the signal of association, and that protein-coding variants in other genes in the *TYK2* locus do not contribute to RA susceptibility.

### Pleiotropic effects of RA-associated *TYK2* variants in other autoimmune diseases

Loci implicated in risk of RA are also associated with risk of other autoimmune diseases [1,22]. *TYK2* protein-coding variants have been associated with several autoimmune diseases, including systemic lupus erythematosus (SLE) and inflammatory bowel disease (IBD) [35–38,40]. In SLE, the reported association is with the common *TYK2* missense variant V362F predicted to be benign (rs2304256, MAF = 23%, OR = 0.70) [35]; in IBD, the reported association is with the *TYK2* variant I684S (OR = 1.12) [36], which we demonstrate as protecting against RA. Accordingly, we explored whether the three RA-associated *TYK2* variants contributed to risk of SLE and IBD or whether the published variants provide a better genetic explanation for the signal of association.

**Fig 4:**
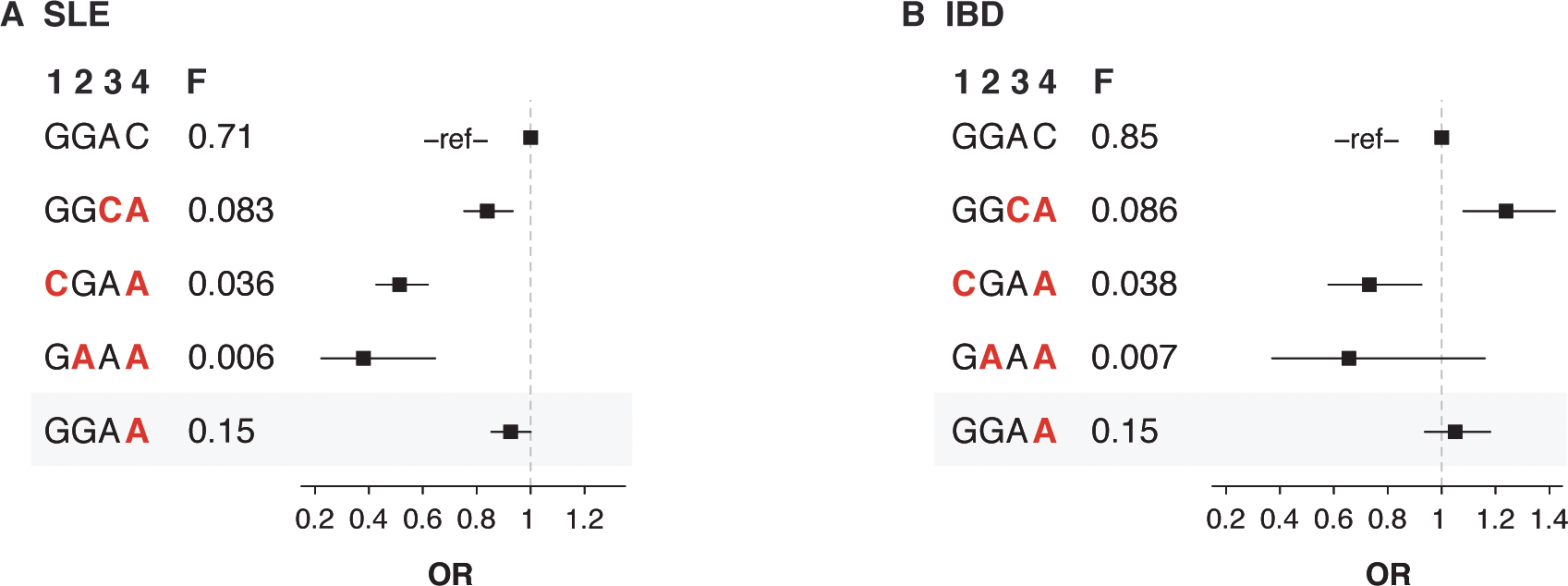
Association of the three RA-associated *TYK2* missense variants with SLE and IBD. We used Exomechip data from 3,053 SLE cases and 13,687 controls (A) and 1,346 IBD cases and 13,687 controls (B) to built haplotypes using the RA-associated *TYK2* variants P1104A, A928V and I684S. In the haplotype model, we also included the *TYK2* SNP V362F, which has previously been reported to be associated with SLE (highlighted in gray). Frequencies of the independent haplotypes and odds ratios (OR) relative to the most frequent haplotype are shown. Minor alleles of the variants are highlighted in red. H, haplotypes; F, haplotype frequency; 1, P1104A; 2, A928V; 3, I684S; 4, V362F.

Using Exomechip genotype data available for 3,053 SLE cases and 13,687 controls of European ancestry, we observed that the three RA-protecting *TYK2* variants P1104A, A928V, and I684S protected against SLE (P_omnibus_ = 6x10^-18^), with effect sizes similar to the effect sizes in RA (Fig 4A). In this dataset, the *TYK2* missense variant V362F previously reported to be associated with SLE [35,37,40] showed a protective effect (OR = 0.85 [0.79–0.92]) at P = 1.8x10^-5^. Importantly, the haplotype analysis highlighted that the V362F association was driven by imperfect LD to the three RA-associated missense variants P1104A, A928V, and I684S (P_omnibus, 3df_ = 6x10^-18^). Indeed, we found no effect of the haplotype carrying only the minor allele of the V362F variant (Fig 4A).

We also analysed Exomechip genotype data available for 1,346 IBD cases and 13,687 controls of European ancestry (Fig 4B). Consistent with previous reports in Crohn’s disease, the *TYK2* I684S variant was associated with increased risk of IBD in our dataset (OR = 1.26 [1.10–1.43], P = 8x10^-4^) [36]. Interestingly, however, we observed a protective effect of the *TYK2* P1104A variant (OR = 0.75 [0.60–0.93], P = 0.008). The point estimate of effect size at the *TYK2* A928V variant was also consistent with protection against IBD (0.64 [0.37–1.1], P = 0.11), although our analysis was underpowered to detect an association at P< 0.05 at this SNP. While additional studies are required to definitively fine-map the *TYK2* locus in IBD (as we have done in RA), our data suggest that *TYK2* protein-coding variants contribute to IBD susceptibility.

Using the Exomechip data available for RA, SLE and IBD, we found no additional *TYK2* variants associated at P<0.05, in either the disease-specific analyses or a diseases-combined analysis (based on the hypothesis that independent genetic variants contribute to susceptibility in all three autoimmune diseases combined) (S2 Fig). Finally, in gene-based association test (SKAT-0), we observed no aggregate signal of association driven by rare *TYK2* protein-coding variants predicted to be damaging, in either SLE or IBD (P>0.05).

### Comprehensive investigation of pleiotropic effects of RA-associated *TYK2* variants using electronic medical records

We next sought to investigate whether the *TYK2* P1104A, A928V and I684S variants protecting against RA and reported or predicted to be LOF were associated with other clinical diagnoses. To that end, we used two independent EMR clinical datasets linked to genotype data: 1) 3,005 individuals of European ancestry from the Informatics for Integrating Biology and the Bedside (i2b2) center [48], and 2) 26,372 individuals of European ancestry from Vanderbilt University Medical Center’s BioVU EMR-linked DNA biobank [49]. We performed an unbiased PheWAS for all common EMR-linked binary traits (N = 502 phenotypes), followed by an analysis focused on clinical traits related to TYK2 biology (N = 30 phenotypes).

**Fig 5:**
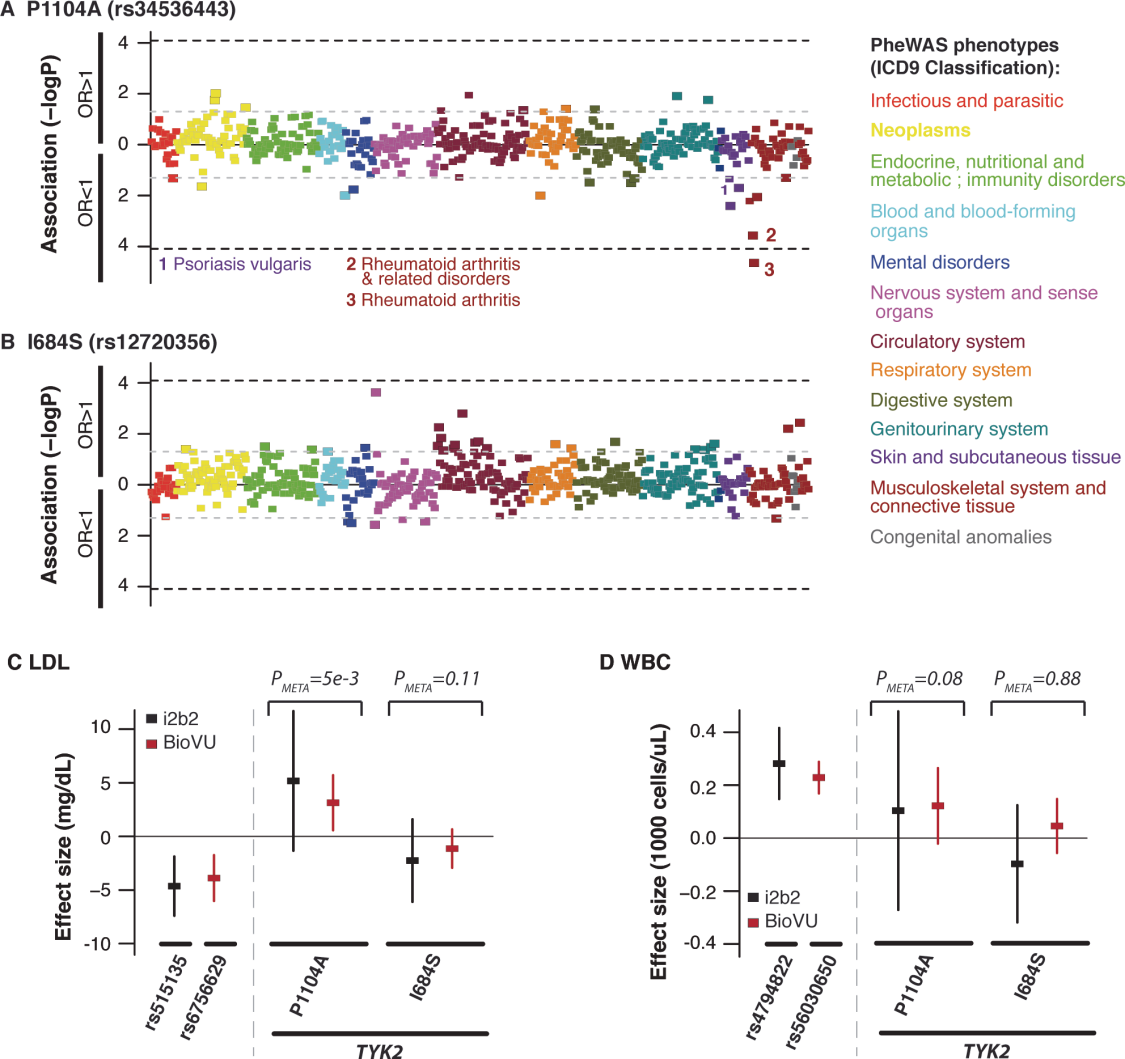
Investigation of pleiotropic effects of RA-protecting *TYK2* variants using electronic medical records. We first tested association of the P1104A (A) and I684S (B) variants to 502 PheWAS phenotypes with frequency>1% in two independent EMR collections including 3,005 and 26,372 individuals of European ancestry, respectively. Pvalues of each PheWAS phenotype in meta-analysis of the two EMR collections are shown. We also tested association of the *TYK2* P1104A and I684S variants with low-density lipoproteins (LDL) levels (C), and white blood cell counts (WBC) (D). Effect sizes and confidence intervals in each EMR collection are shown. Pvalues from meta-analysis of the two EMR collections are indicated. Association results from SNPs previously reported to be associated with each quantitative trait (indicated by their respective rsIDs) are also shown.

In the PheWAS testing the association between the *TYK2* variants and the 502 common binary phenotypes (phenotype frequency>1%), we only observed a significant association (P<1x10^-4^) between the P1104A variant and RA (OR = 0.65, P_META_ = 2.3x10^-5^), with an effect size consistent to the effect size observed in the Immunochip RA case-control cohort (Fig 5A) [2]. We observed no PheWAS phenotype associated with the I684S or A928V variants at P_META_<10^–4^ (Fig 5B and S3 Fig). (The 502 PheWAS phenotypes tested did not include SLE and IBD, which both had a frequency <1% in the i2b2 collection.) The list of PheWAS phenotypes associated at P_META_<0.05 with consistent effect sizes in both EMR datasets (with P_META_<P _BioVU_ and P_META_<P_i2b2_) is shown in S7 Table. Together, these results provided no evidence of association with strong increased risk (OR≥1.3, based on our power calculations; S4–S6 Figs) between the RA-protecting *TYK2* variants and any of the PheWAS phenotypes tested.

Complete LOF of TYK2 leads to human primary immunodeficiency, which is caused by rare autosomal recessive null mutations in *TYK2* and results in increased risk of severe infections (bacterial, viral and fungal) [50,51]. To investigate the hypothesis that individuals carrying the P1104A, A928V or I684S variants might be at increased risk of serious infection due to partial inhibition of TYK2, we used a comprehensive set of infection-related ICD9 codes developed and validated elsewhere [52,53]. We found no group of infection significantly associated with either of the P1104A, A928V or I684S variants (P<1x10^-4^) (S8 Table). We observed suggestive evidence of association between the A928V variant and increased risk of pneumonia (OR = 1.48, P = 0.011 in the BioVU dataset; OR = 2.59, P = 0.079 in the i2b2 dataset; OR_META_ = 1.54, P_META_ = 0.004 in meta-analysis), but the signal did not surpass significance thresholds after multiple hypotheses testing correction

Finally, we tested the association of the P1104A, A928V and I684S variants with two quantitative traits available in the EMR: white blood cell counts (WBC) and low-density lipoprotein (LDL) levels (Fig 5C and 5D, S3 Fig, and S9 Table). We selected these two phenotypes as a drug, tofacitinib, targets a pathway related to TYK2—the JAK signalling pathway—and patients treated with JAK-inhibitors have lower levels of WBCs/neutrophils and elevated levels of LDL cholesterol [54,55]. We observed only suggestive association of the *TYK2* P1104A variant with increased LDL levels (BETA_META_ = +3.4 mg/dL, P_META_ = 0.005 in the meta-analysis) (Fig 5C and S9 Table) and no association of the *TYK2* variants with WBC. As a positive control, we showed significant association to WBC and LDL levels of known associated SNPs from previous GWAS investigating these two traits [56–60], demonstrating that our analysis using EMR had the power to detect associations to these traits (Fig 5C and 5D, and S10 Table).

## Discussion

Previous studies had identified one *TYK2* missense variant, P1104A (rs34536443), as associated with RA susceptibility [2]. Here, through dense genotyping, haplotype analyses and deep sequencing, we demonstrate that three independent *TYK2* missense variants (P1104A, A928V [rs35018800] and I684S [rs12720356]) unequivocally protect against RA (Figs 1, 2, and 3). In aggregate, the 3 *TYK2* variants account for 0.25% of the phenotypic variance of RA. Together with the lack of convincing association to protein-coding variants in *ICAM* genes and other genes from the 19p32.3 locus, our results provide multiple lines of evidence implicating *TYK2*, rather than another nearby gene, as a causal gene involved in RA disease susceptibility.

TYK2 is a member of the JAK family. The four JAK proteins (JAK1, JAK2, JAK3 and TYK2) selectively associate with various cytokine receptors [30,31]. While JAK1 and JAK2 have broad functions, JAK3 and TYK2 are primarily important for immune responses. TYK2 associates with receptor chains utilized by a large number of cytokines, including IL6-R, which is the target of tocilizumab, an anti-IL6R monoclonal antibody used in the treatment of RA [32,61]. Two of the variants associated with RA in our study, P1104A and I684S, have recently been shown to affect TYK2 function in primary T cells, fibroblasts and B cell lines and impair pro-inflammatory cytokines signalling, providing evidence that both variants are LOF mutations and that LOF mutations in *TYK2* alter immune-mediated pathways [33,34].

We have previously proposed three features of human genetics that can be applied to drug discovery [20]: (1) identification of targets that, when perturbed in a manner that mimics trait-associated alleles, demonstrate efficacy in treating complex human diseases—as illustrated by recent studies highlighting the increased success rate of targets supported by human genetics [19]; (2) identification of alternative clinical indications through genetic associations of related diseases for drug repurposing [62]; and (3) prediction of potential on-target adverse drug events via pleiotropic associations [7,63]. In the present study, we explored all three features as it pertains to the *TYK2* locus.

First, the observation of multiple independent RA-protecting variants (Figs 1, 2, and 3) provides an accumulation of evidence that a drug that mimics the effect of *TYK2* alleles may be effective at treating RA (proxy for drug efficacy). This concept is consistent with the overlap between human genetics and drug discovery in other diseases [62], as exemplified by Mendelian randomization studies on variants in *PCSK9* and *IL6R* [7,20,63]. In addition, the recent development of drugs inhibiting the TYK2-related proteins JAK1, JAK2 and JAK3 for the treatment of RA (including the drug tofacitinib, recently approved by the food and drug administration [FDA]), further support TYK2 as an appealing candidate drug target [54,64].

Second, the protective effect of the three *TYK2* variants in SLE observed in our study (Fig 4A) highlights that a drug that mimics the effect of the RA-protecting *TYK2* alleles may also be effective at treating SLE, and potentially other autoimmune diseases such as IBD. We note that the relatively small sample size in IBD (n = 1,346 IBD cases) limits our ability to perform detailed fine-mapping of the *TYK2* locus in IBD, and that additional studies are required.

As a third feature, we used electronic medical records to comprehensively investigate pleiotropy of RA-associated *TYK2* variants that could predict potential adverse events, including risk of serious infections, decreased WBC or neutrophil counts, or increased LDL levels, which are major adverse drug events in RA drug development that have been observed in clinical trials of tofacitinib [54,55,64] (Fig 5 and S3 Fig). In this analysis, we observed no strong evidence of a phenotype at increased risk in carriers of the *TYK2* RA protecting-variants P1104A, A928V or I684S. However, we did observe several phenotypes with suggestive evidence of association (P_META_<0.05) and consistent direction of effect in two independent EMR datasets (Fig 5, S7 and S8 Tables), including association of the P1104A variant with increased LDL levels (Beta = +3.4 mg/dL, P_META_ = 0.005), and association of the rare A928V variant with risk of pneumonia (OR = 1.5, P_META_ = 0.004). These observations will require further investigation in very large collections to predict whether serious infections and/or hypercholesterolemia might be a potential on-target adverse event of a drug mimicking the effect of these alleles.

There are limitations to our study. It is possible that RA-associated *TYK2* variants have pleiotropic associations with other phenotypes (e.g., infection), but that our EMR-based approach was not able to detect these associations at the level of statistical significance in our study. For example, EMR diagnostic codes are comprehensive but imprecise, which limits accurate estimations of effect size of associations from EMR data alone. Further, our EMR analysis had limited power to identify individual diagnoses associated with *TYK2* LOF, considering the sample size and prevalence of the individual diagnoses. However, the use of EMR has been shown to successfully detect associations with a large spectrum of phenotypes [25]. In the present study, we were able to replicate previously published associations (Fig 5 and S10 Table). Of note, GWAS have not yet proven to be successful at identifying loci contributing to risk of infection with reproducible results, which limits our ability to include infection-related variants as a positive control in our PheWAS.

In conclusion, although previous studies have nominated TYK2 as a potential therapeutic target [41,65], our study provides compelling human genetic data demonstrating that *TYK2* alleles with partial loss-of-function (1) protect against RA, SLE and potentially other autoimmune diseases such as IBD; and (2) are tolerated in the general population, as there are no obvious detrimental associations in our PheWAS. Our results also highlight the potential of investigating in details the different biological effects of the three *TYK2* variants to inform drug efficacy, toxicity and repurposing at early stages of drug development. In theory, the same approach linking human genetics with “real life data” like EMR should be applicable to other complex diseases, thereby providing an estimate of drug efficacy and toxicity at the time of target validation.

## Methods

### Samples and ethics statement

A detailed description of the samples included in this study is provided in S1 and S2 Tables, and in the related Methods sections. Our study was approved by the Institutional Review Board of Brigham & Women's Hospital. All the enrolled subjects provided written informed consent for the participation of the study. Blood samples were collected according to protocols approved by local institutional review boards.

### RA case-control Immunochip dataset

To fine-map the *TYK2* locus and investigate independent signals of association to RA, we used 7,222 ACPA+ RA cases and 15,870 controls genotyped on the Immunochip platform as part of the Rheumatoid Arthritis Consortium International (S1 Table) [2]. Quality control and initial data filtering were performed as described previously [2]. Briefly, genotype calling was performed on all samples as a single project using the GenomeStudio Data Analysis software. SNPs with low cluster separation, call rate <0.99, or departure from Hardy-Weinberg equilibrium (*P*_HWE_ < 5.7x10^−7^) were excluded from each of the collections. Samples with a call rate <0.99 were excluded. Principal component analysis (PCA) was performed using EIGENSOFT v4.2 [66] with HapMap phase 2 samples as reference populations, and non-Caucasians samples were excluded. A second PCA was performed to exclude outliers and calculate the principal components (PCs) to include as covariates in the logistic regressions. We build haplotypes using BEAGLE, and tested for association of the genotypes and haplotypes with risk of RA, using PLINK (including 10 PCs as covariates) [67,68].

### Autoimmune diseases—case control Exomechip datasets

To replicate association signals to RA, we used an unpublished dataset of 4,726 RA cases and 13,683 controls genotyped on the Exomechip (S1 Table). This dataset included samples collected from the Dutch Rheumatoid Arthritis Monitoring (DREAM) registry [69], the Informatics for Integrating Biology and the Bedside (i2b2) cohort [48], the North American Rheumatoid Arthritis Consortium (NARAC) family cohort [70], the Study of New Onset Rheumatoid Arthritis (SONORA) cohort [71], the Veteran's Affairs Rheumatoid Arthritis registry (VARA) [72].

We also tested the association of *TYK2* variants to SLE and IBD using Exomechip genotype data from 3,053 SLE cases and 1,346 IBD cases (S1 Table). The SLE dataset included samples from the Autoimmune Biomarkers Collaborative Network (ABCoN) [73], Genentech Clinical Trials, the Multiple Autoimmune Disease Genetics Consortium (MADGC) [74], the Oklahoma Medical Research Foundation (OMRF) [75], the University of California San Francisco (UCSF) [76], and the UK King’s College [77]. The IBD dataset included samples from the University of Dundee [78], the EMerging BiomARKers in Inflammatory Bowel Disease (EMBARK) study [79], and Genizon BioSciences, Inc [80].

Extensive quality control and data filtering were performed in each dataset. Samples were selected by excluding: 1) individuals with <95% complete Exomechip genotype data; 2) individuals with IBD [Pi-hat >0.125 using 10,000 higher frequency SNPs (MAF>0.05)], 3) non-European ancestry (>0.10, based on STRUCTURE (v2.3.3) [81] analyses using core sets of different continental groups including >100 subjects from each ancestry (European, East Asian, Amerindian, South Asian and West African) and genotypes from >2000 high frequency LD independent SNPs; and 4) PCA outliers (>5 SD for first 10 principal components). The PCA was performed in EIGENSOFT 4.2 [66] using 13,682 Exomechip SNPs that 1) were not in LD (r^2^<0.01); 2) >99% complete typing data and 3) were enriched for SNPs with minor allele frequencies >0.05 (>50% of SNPs). Independence of the samples from the Exomechip and Immunochip RA datasets was confirmed using overlapping SNPs.

We build haplotypes using BEAGLE, and tested for association of the genotypes and haplotypes with risk of RA, SLE and IBD, using PLINK (including 10 PCs as covariates) [67,68].

### Exon sequencing

To investigate the contribution to RA of rare protein-coding variants at the *TKY2* locus, we used exon-sequencing data available in 1,420 RA patients and 1,340 controls originating from Europe or the United States (S2 Table). A total of 10 collections from 5 countries were included: the Autoimmune Biomarkers Collaborative Network (ABCoN) [82], the Academic Medical Center (AMC) and VU University medical center (VUMC), the UK Biological in Rheumatoid arthritis Genetics and Genomics Study Syndicate (BRAGGSS) [83], the Consortium of Rheumatology Researchers of North America (CORRONA) [84], the Informatics for Integrating Biology and the Bedside (i2b2) center, the Leiden University Medical Center (LUMC) [85], the Dutch Rheumatoid Arthritis Monitoring registry (DREAM) and the Nijmegen Biomedical Study (NBS) [69], the French Research in Active Rheumatoid Arthritis (ReAct) [86], and the Rheumatic Diseases Portuguese Registry (Reuma.pt/ Biobanco-IMM) [87]. DNA libraries were prepared in sets of 96. The barcoded libraries from each set were then pooled together. Enrichment of the target genomic regions was performed using the NimbleGen Sequence Capture technology. After target capture, each pool was loaded on two lanes of the HiSeq sequencer. Reads were then aligned to the reference human genome (NCBI Build37/hg19) using BWA [88] and duplicate reads were excluded using Picard. In total, 95% of the samples reached an minimum average coverage of 20X in >70% of target regions, with 96% of the target regions in the samples passing this initial quality control (QC) covered at > = 20X coverage. Single nucleotide polymorphisms (SNPs) were called using Samtools v1.16 [89] and VarScan 2.2.9 [90] using stringent minimum coverage, mapping quality, and strandness filters. SNPs called from each sample using both calling algorithms were then merged and additional filters were applied (number and frequency of the reads supporting the variant, position in the reads). Finally, only variants passing filters in >50% of the samples were considered high-quality and included in the subsequent analysis. Sequencing, initial QC and SNP calling were performed at the Genome Institute. After applying stringent filters to remove individuals based on sequencing coverage and quality (N = 131 individuals excluded), and population stratification using case-control principal components (PC)-matching (N = 393 individuals excluded), a final set of 1,118 case-control matched pairs of European ancestry was included in the association tests (S1 Table). The transition:transversion ratio based on the variants passing QC was 2.5 (vs 2.6 for dbSNP SNPs in target space). MAF correlation of variants called in the sequenced controls and samples from the Exome Sequencing Project (ESP) was 98%. Concordance between sequencing genotype calls and Exomechip data available for 137 samples was calculated to further assess the quality of the sequencing data. Overall, we observed 99.7% concordance at 1,718 shared variants polymorphic in the 137 samples set. The variants were annotated using ANNOVAR [91]. We used PolyPhen-2 and SIFT to predict the function of the missense variants [43,44]. We then grouped the variants based on the prediction results from both software: 1) benign in both PolyPhen-2 and SIFT (that we considered as benign), 2) benign using one software and possibly/probably damaging using the other software (that we considered as benign), 3) possibly damaging in both PolyPhen-2 and SIFT or possibly damaging in one software and probably damaging using the second software (that we considered as “potentially damaging”), 4) probably damaging in both PolyPhen-2 and SIFT (that we considered as “potentially damaging”).

### Gene-based association tests

We performed gene-based association tests to investigate the contribution of rare variants (MAF<0.5%) to protection from RA. For each of the 11 genes in the *TYK2* locus (defined by the SNPs in linkage disequilibrium (LD, r^2^>0.5) to the strongest association to RA (driven by rs34536443, P1104A) [2], we investigated the overall contribution of 1) all rare (MAF<0.5%) missense variants, and 2) the rare nonsense variants and the missense variants predicted to be “potentially damaging” using Polyphen-2 and SIFT [43,44]. We used 3 published “one-sided” methods: (1) the classic burden test, (2) the frequency-weighted (FW) test and (3) the Variable-threshold (VT) test, all 3 tests implemented in PLINKSEQ [46,47]. We also used SKAT-O (“two-sided” method) [45]. We performed all 4 tests with 10,000 case-control permutations to assess empirical P-values.

In addition to the gene-based tests, we performed window-based tests to investigate the contribution of rare variants per 500 bp window of the genes coding sequence, using SKAT-O. and 10,000 case-control permutations to assess empirical P-values. Finally, we used the one-sided methods (BURDEN, WT, VT) to further assess the contribution of rare variants in the protein kinase 1 domain of *TYK2* in domain-based tests.

### Linking haplotypes with clinical diagnoses from Electronic Medical Records

To comprehensively investigate pleiotropic effects of *TYK2* LOF variants, we used two independent electronic medical records (EMR) datasets linked to genotype data: 1) EMR data from 3,005 individuals of European ancestry from the Informatics for Integrating Biology and the Bedside (i2b2) center who received medical care within the Brigham and Women’s Hospital (BWH) and Massachusetts General Hospital (MGH) healthcare system linked to Immunochip genotype data [48,92], and 2) EMR data from 26,372 individuals of European ancestry from BioVU, the Vanderbilt University DNA biobank [49], linked to Exomechip genotype data (S1 Table). The i2b2 collection was initially optimized for RA genetic studies [48,92], resulting in a high frequency of patients with ICD9 code = 714 (Rheumatoid arthritis and other inflammatory polyarthropathies) and 714.0 (Rheumatoid arthritis) in this collection (S1 Table). The BioVU Exomechip cohort was primarily chosen based on longitudinal exposure in healthcare system, without a specific emphasis on RA or other autoimmune diseases. *International Classification of Diseases 9th Revision* (ICD9) codes were grouped into 1,570 clinically relevant phenotypes using the current version of the PheWAS codes, as described previously [25]. We restricted our analysis to PheWAS codes referring to ICD9 001–779 (ignoring signs and symptoms and injuries) and with a prevalence >1% in both EMR datasets, resulting in 502 PheWAS codes. For each PheWAS code, we considered individuals with at least two reported events as cases.

We first conducted a phenome-wide association study (PheWAS). In each EMR dataset, we tested for association of *TYK2* variants with each PheWAS code in the additive model using logistic regressions adjusted for age, gender and PCs to correct for population stratification. In the analysis of the i2b2 EMR dataset, we further adjusted for RA status. We conducted an inverse-variance-weighted meta-analysis to combine the results from the two EMR datasets.

We then conducted an association study focused on ICD9 codes related to serious infections. We used two previously published sets of ICD9 codes for serious infections grouped by anatomical site and compiled based on expert consensus: 1) a ‘‘comprehensive” set that included a wide range of codes to maximize sensitivity; 2) a ‘‘restricted” set including more specific ICD9 codes [53].

Finally, we tested for association of the *TYK2* variants with two quantitative traits available in the EMR: white blood cell counts (WBC), and low-density lipoprotein (LDL) levels. For WBC, we defined the primary outcome as the mean of all measurements available for each subject in the EMR. We tested the association between the *TYK2* variants and the mean values, adjusted by age, gender, PCs and the number of measurements used to calculate the mean values. For LDL levels, the primary outcome was defined by each subject’s first LDL measurement in the EMR as described previously [93]. We excluded subjects with electronic prescription for an HMG-CoA reductase inhibitor (statin) prior to the LDL measurement to maximize the chance of selecting subjects prior to any lipid lowering intervention. We tested the association between the *TYK2* variants and the first LDL measurement, adjusted by age at LDL measurement, gender and PCs. We also adjusted for year of LDL measurement, which has been shown to strongly contribute to the variability in LDL levels [93,94].

### Estimation of the statistical power in the PheWAS

To estimate the power to detect a significant association (at P<1x10^-4^) in the PheWAS based on the sample size, the frequency of each code and the frequency of the SNPs tested, we used and R script adapted from the ldDesign R package to query the Genetic Power Calculator online tool [95]. We assessed power for a variant with MAF = 3.4% (corresponding to the P1104A variant MAF), MAF = 8.7% (corresponding to the I684S variant MAF) and MAF = 0.7% (corresponding to the A928V variant MAF). We assessed power for a set of code frequencies and ORs, in the models where 1) the RA-protecting variants increase risk (risk allele frequency [RAF] = 0.034, RAF = 0.087 and RAF = 0.007) or decrease risk (RAF = 0.966, RAF = 0.913 and RAF = 0.993).

Based on the case:control ratio for each phenotype, the PheWAS approach has significantly greater power to detect increased risk compared to protection (S4–S6 Figs). Estimations highlighted the statistical power to detect a significant association (P<1x10^-4^; P = 0.05/532 phenotypes tested) to the P1104A and I684S variants. For rs34536443, we estimated power to detect a significant association with: 1) OR≥2 at phenotype frequency = 1%, 2) OR≥1.4 at phenotype frequency = 10% in BioVu. For rs12720356, we estimated power to detect a significant association with: 1) OR≥1.6 at phenotype frequency = 1%, OR≥1.3 at phenotype frequency = 10% in BioVU (S4 and S5 Figs). However, we estimated limited power to detect a significant association to the rare variant A928V (MAF = 0.7%; S6 Fig).

## Supporting Information

S1 Fig Sliding-window test results using exon-sequencing of RA cases and controls. An accumulation of true rare missense variants (MAF<0.5%) predicted to be damaging was observed in the Protein kinase 1 domain of *TYK2*. Association results from 500 bp sliding window tests in SKAT-O restricted to nonsense variants (pink) and missense variants predicted to be damaging (red) are shown. Variants with MAF>1% (indicated by a star) were excluded in the test. In TYK2, we further excluded the A928V and A53T variants with 0.5%<MAF<1% (indicated by a star) that were independently investigated using Exomechip data. The light blue background highlights the coding sequence region with P<0.05.
(PDF)

S2 Fig Association to RA, SLE and IBD of all TYK2 variants genotyped on the Exomechip and predicted to be damaging. Only the 3 variants with MAF>0.5% confirmed to be associated to RA in our study reached P<0.05, in either the disease-specific analyses or the diseases-combined analysis.
(PDF)

S3 Fig Investigation of pleiotropic effects of TYK2 A928V variant (rs35018800) using electronic medical records. (A) We first tested association of the A928V variant to 502 PheWAS phenotypes with frequency>1% in two independent EMR collections. Pvalues of each PheWAS phenotype in meta-analysis of the two EMR collections are shown. We also tested association of the A928V variant with LDL levels (B), and white blood cell counts (WBC) (C). Effect sizes and confidence intervals in each EMR collection are shown.
(PDF)

S4 Fig Estimation of power to detect an association at rs34536443 in the EMR. We estimated the power to detect an association at P<1x10^-4^ for a variant with MAF = 3.4%, based on phenotype frequency in the EMR and estimated OR. (A) Power estimations for a sample size of 3,005 subjects. (B) Power estimations for a sample size of 26,372 subjects. The left panel shows results for the minor allele associated with increased risk. The right panel shows results for the minor allele with a protective effect. The dashed red line indicated a phenotype frequency of 1%. The barplots highlight the number of cases per phenotype in the EMR collections.
(PDF)

S5 Fig Estimation of power to detect an association at rs12720356 in the EMR. We estimated the power to detect an association at P<1x10^-4^ for a variant with MAF = 8.5%, based on phenotype frequency in the EMR and estimated OR. (A) Power estimations for a sample size of 3,005 subjects. (B) Power estimations for a sample size of 26,372 subjects. The left panel shows results for the minor allele associated with increased risk. The right panel shows results for the minor allele with a protective effect. The dashed red line indicated a phenotype frequency of 1%. The barplots highlight the number of cases per phenotype in the EMR collections.
(PDF)

S6 Fig Estimation of power to detect an association at rs35018800 in the EMR. We estimated the power to detect an association at P<1x10^-4^ for a variant with MAF = 0.7%, based on phenotype frequency in the EMR and estimated OR. (A) Power estimations for a sample size of 3,005 subjects. (B) Power estimations for a sample size of 26,372 subjects. The left panel shows results for the minor allele associated with increased risk. The right panel shows results for the minor allele with a protective effect. The dashed red line indicated a phenotype frequency of 1%. The barplots highlight the number of cases per phenotype in the EMR collections.
(PDF)

S1 Table Description of the subjects included in this study. (PDF)

S2 Table Meta-analysis of Immunochip, Exomechip and Sequencing RA association results. (PDF)

S3 Table Detailed description of the samples including in the sequencing study. (PDF)

S4 Table Gene-based association results, restricted to nonsense and missense variants with MAF<0.5% and predicted to be possibly or probably damaging in PolyPhen-2 and SIFT. (PDF)

S5 Table Description of the 26 missense variants predicted to be damaging identified in *TYK2* by sequencing of 1,118 RA cases and 1,118 controls. (PDF)

S6 Table Domain-based association results, restricted to missense variants in the protein kinase 1 domain-coding region of *TYK2* with MAF<0.5% and predicted to be possibly or probably damaging. (PDF)

S7 Table Results of association between RA-protecting *TYK2* variants and PheWAS phenotypes with consistent effect sizes in the two independent EMR datasets and P<0.05 in the meta-analysis. (PDF)

S8 Table Results of association between RA-protecting *TYK2* variants and clinical diagnoses related to serious infections. (XLS)

S9 Table Results of association between RA-protecting *TYK2* variants and two quantitative traits: low-density lipoproteins levels and white blood cell counts. (XLS)

S10 Table Association results of SNPs reported to be associated with lipid levels and white blood cell counts and genotyped in the BioVU or in the i2b2 EMR collection. (PDF)
