# Supplementary material for: TYK2 Protein-Coding Variants Protect against Rheumatoid Arthritis and Autoimmunity, with No Evidence of Major Pleiotropic Effects on Non-Autoimmune Complex Traits
Source: PLoS One. 2015 Apr 7;10(4):e0122271. doi: 10.1371/journal.pone.0122271 (PMC4388675; doi:10.1371/journal.pone.0122271)
Supplement: S1 Fig — An accumulation of true rare missense variants (MAF<0.5%) predicted to be damaging was observed in the Protein kinase 1 domain of TYK2. Association results from 500 bp sliding window tests in SKAT-O restricted to nonsense variants (pink) and missense variants predicted to be damaging (red) are shown. Variants with MAF>1% (indicated by a star) were excluded in the test. In TYK2, we further excluded the A928V and A53T variants with 0.5%<MAF<1% (indicated by a star) that were independently investigated using Exomechip data. The light blue background highlights the coding sequence region with P<0.05. (PDF) [file pone.0122271.s001.pdf]

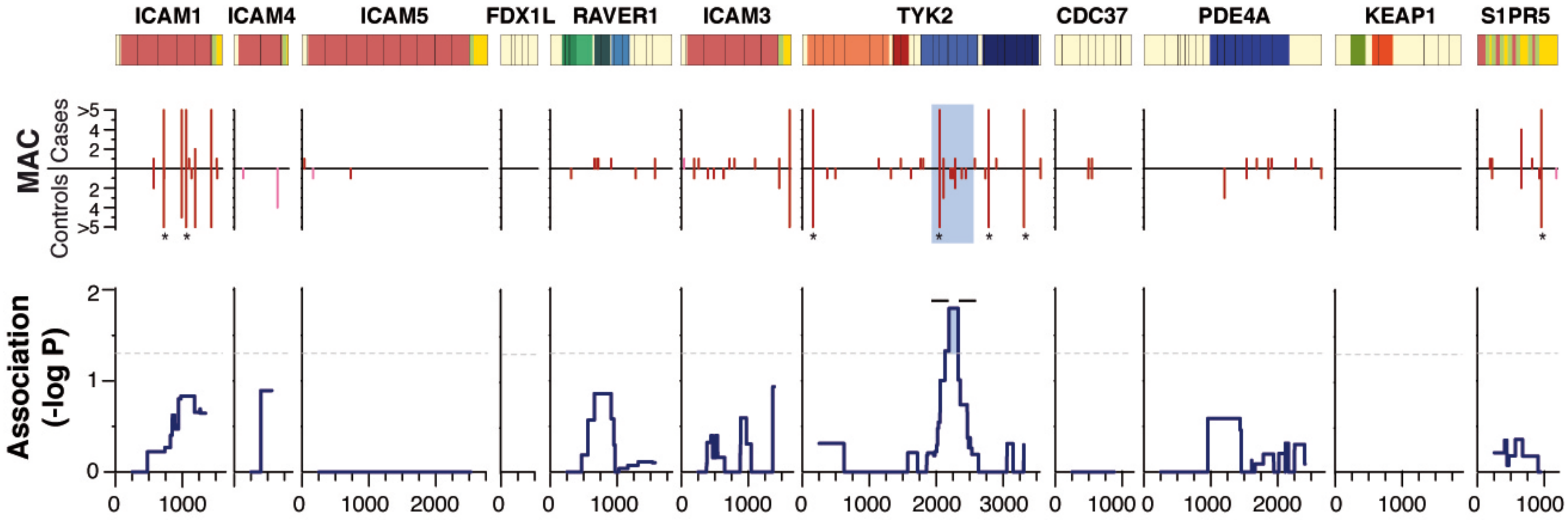

S1 Fig. Sliding-window test results using exon-sequencing of RA cases and controls. An accumulation of true rare missense variants (MAF<0.5%) predicted to be damaging was observed in the Protein kinase 1 domain of *TYK2*. Association results from 500 bp sliding window tests in SKAT-O restricted to nonsense variants (pink) and missense variants predicted to be damaging (red) are shown. Variants with MAF>1% (indicated by a star) were excluded in the test. In TYK2, we further excluded the A928V and A53T variants with 0.5%<MAF<1% (indicated by a star) that were independently investigated using Exomechip data. The light blue background highlights the coding sequence region with P<0.05.
