# Supplementary material for: TYK2 Protein-Coding Variants Protect against Rheumatoid Arthritis and Autoimmunity, with No Evidence of Major Pleiotropic Effects on Non-Autoimmune Complex Traits
Source: PLoS One. 2015 Apr 7;10(4):e0122271. doi: 10.1371/journal.pone.0122271 (PMC4388675; doi:10.1371/journal.pone.0122271)
Supplement: S2 Fig — Only the 3 variants with MAF>0.5% confirmed to be associated to RA in our study reached P<0.05, in either the disease-specific analyses or the diseases-combined analysis. (PDF) [file pone.0122271.s002.pdf]

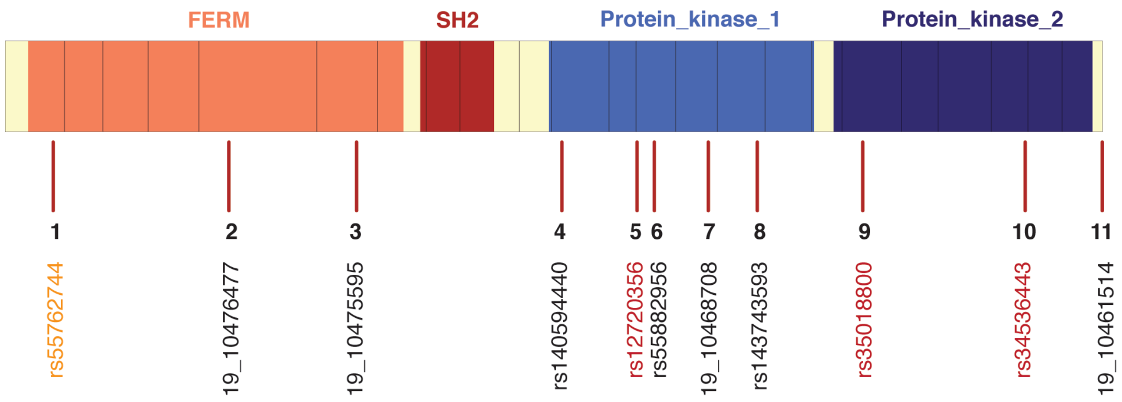

	1	2	3	4	5	6	7	8	9	10	11
**SNP**	**rs55762744**	**19_10476477**	**19_10475595**	**rs140594440**	**rs12720356**	**rs55882956**	**19_10468708**	**rs143743593**	**rs35018800**	**rs34536443**	**19_10461514**
**Substitution**	**A53T**	**R243W**	**R381W**	**V603M**	**I684S**	**R703W**	**G761V**	**P814L**	**A928V**	**P1104A**	**C1187Y**
MAF (%)											
RA	1,11	0	0,032	0,021	7,66	0,13	0	0,042	0,56	3,13	0
SLE	0,88	0,016	0,016	0,016	7,57	0,066	0,016	0,033	0,28	2,46	0
IBD	0,82	0	0	0	10,45	0,074	0	0	0,52	3,38	0
Controls	1,07	0,0073	0,084	0,026	8,49	0,14	0,004	0,011	0,76	4,35	0,011
Association in RA *(4,726 cases, 13,683 controls)*											
OR	0.98	NA	0.72	0.71	**0.89**	0.99	NA	4.19	**0.69**	**0.70**	NA
SE	0.12	NA	0.62	0.83	0.046	0.34	NA	0.79	0.16	0.067	NA
P	0.87	NA	0.60	0.67	**0.012**	0.97	NA	0.07	**0.019**	**6.0e-8**	NA
Association in SLE *(3,053 cases, 13,687 controls)*											
OR	0.81	2.11	0.23	0.81	0.88	0.47	4.01	2.91	**0.37**	**0.55**	NA
SE	0.15	1.26	1.03	1.07	0.054	0.53	1.42	0.92	0.25	0.09	NA
P	0.15	0.55	0.15	0.84	**0.014**	0.15	0.32	0.24	**8.1e-5**	**1.5e-11**	NA
Association in IBD *(1,346 cases, 13,687 controls)*											
OR	0.74	NA	NA	NA	**1.26**	0.51	NA	NA	0.64	**0.75**	NA
SE	0.22	NA	NA	NA	0.07	0.73	NA	NA	0.28	0.11	NA
P	0.17	NA	NA	NA	**0.0008**	0.35	NA	NA	0.11	**0.008**	NA
Association in 3 autoimmune diseases combined *(9,125 cases, 13,683 controls)*											
OR	0.88	0.72	0.43	0.66	0.94	0.75	1.17	2.96	**0.58**	**0.65**	NA
SE	0.10	1.23	0.55	0.70	0.04	0.29	1.42	0.71	0.13	0.05	NA
P	0.20	0.79	0.12	0.55	0.070	0.32	0.91	0.13	**2.5e-5**	**1.1e-15**	NA

S2 Fig. Association to RA, SLE and IBD of all TYK2 variants genotyped on the Exomechip and predicted to be damaging. Only the 3 variants with MAF>0.5% confirmed to be associated to RA in our study reached P<0.05, in either the disease-specific analyses or the diseases-combined analysis.
