# Supplementary material for: TYK2 Protein-Coding Variants Protect against Rheumatoid Arthritis and Autoimmunity, with No Evidence of Major Pleiotropic Effects on Non-Autoimmune Complex Traits
Source: PLoS One. 2015 Apr 7;10(4):e0122271. doi: 10.1371/journal.pone.0122271 (PMC4388675; doi:10.1371/journal.pone.0122271)
Supplement: S3 Fig — (A) We first tested association of the A928V variant to 502 PheWAS phenotypes with frequency>1% in two independent EMR collections. Pvalues of each PheWAS phenotype in meta-analysis of the two EMR collections are shown. We also tested association of the A928V variant with LDL levels (B), and white blood cell counts (WBC) (C). Effect sizes and confidence intervals in each EMR collection are shown. (PDF) [file pone.0122271.s003.pdf]

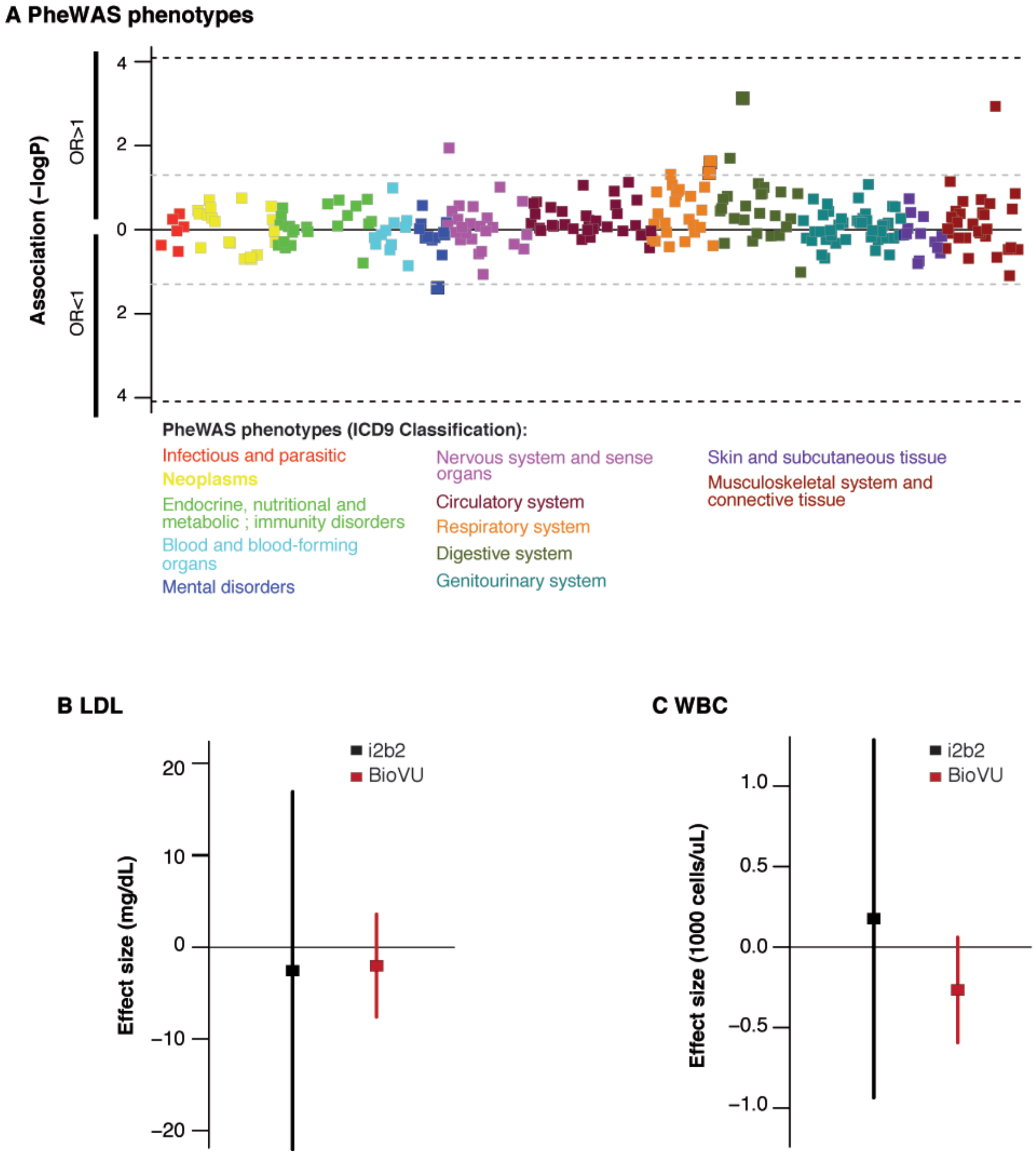


**S3 Fig. Investigation of pleiotropic effects of TYK2 A928V variant (rs35018800) using electronic medical records.** (A) We first tested association of the A928V variant to 502 PheWAS phenotypes with frequency>1% in two independent EMR collections. Pvalues of each PheWAS phenotype in meta-analysis of the two EMR collections are shown. We also tested association of the A928V variant with LDL levels (B), and white blood cell counts (WBC) (C). Effect sizes and confidence intervals in each EMR collection are shown.
