# Supplementary material for: TYK2 Protein-Coding Variants Protect against Rheumatoid Arthritis and Autoimmunity, with No Evidence of Major Pleiotropic Effects on Non-Autoimmune Complex Traits
Source: PLoS One. 2015 Apr 7;10(4):e0122271. doi: 10.1371/journal.pone.0122271 (PMC4388675; doi:10.1371/journal.pone.0122271)
Supplement: S5 Fig — We estimated the power to detect an association at P<1x10^-4^ for a variant with MAF = 8.5%, based on phenotype frequency in the EMR and estimated OR. (A) Power estimations for a sample size of 3,005 subjects. (B) Power estimations for a sample size of 26,372 subjects. The left panel shows results for the minor allele associated with increased risk. The right panel shows results for the minor allele with a protective effect. The dashed red line indicated a phenotype frequency of 1%. The barplots highlight the number of cases per phenotype in the EMR collections. (PDF) [file pone.0122271.s005.pdf]

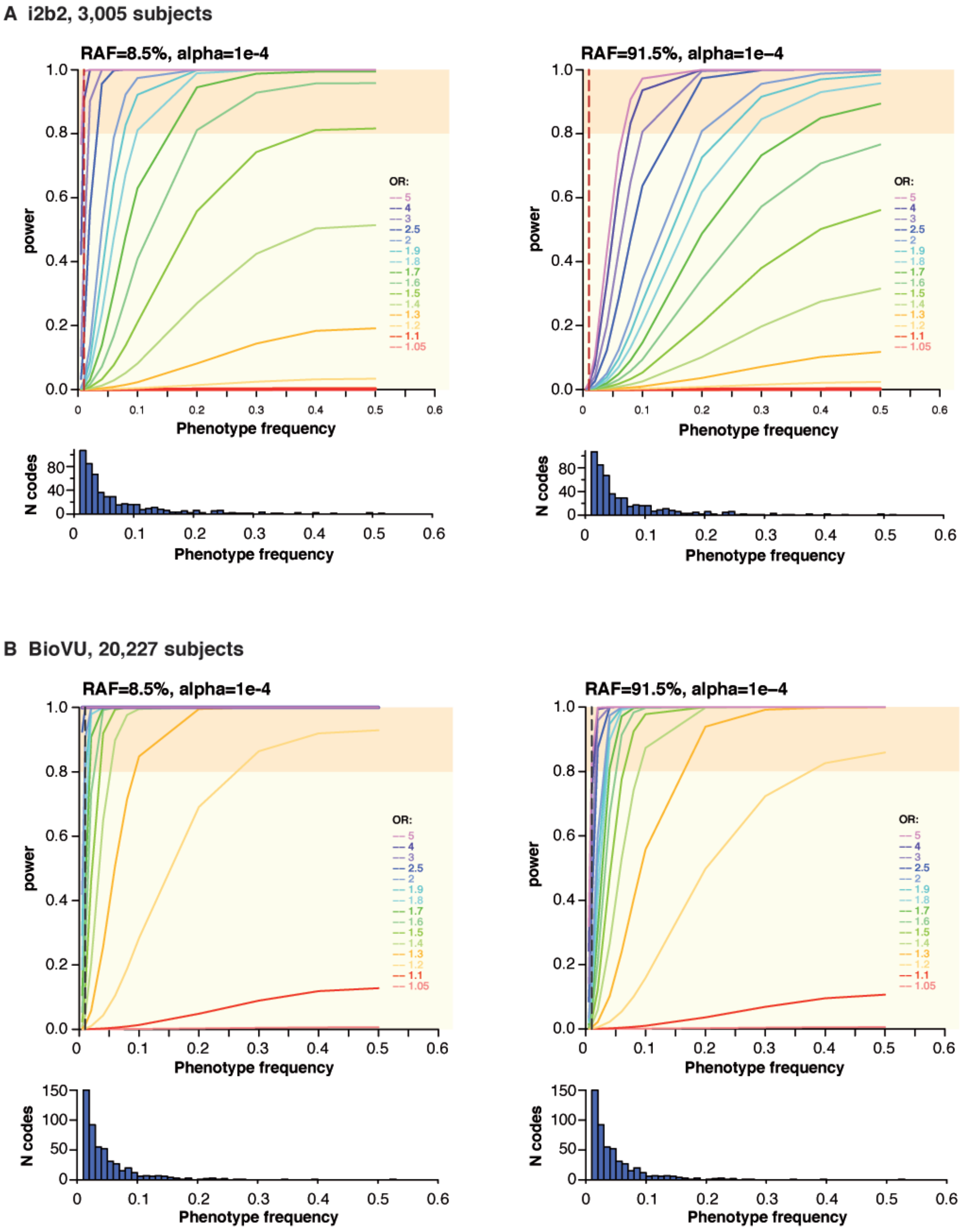

S5 Fig. Estimation of power to detect an association at rs12720356 in the EMR. We estimated the power to detect an association at P<1x10^{-4} for a variant with MAF =8.5%, based on phenotype frequency in the EMR and estimated OR. (A) Power estimations for a sample size of 3,005 subjects. (B) Power estimations for a sample size of 26,372 subjects. The left panel shows results for the minor allele associated with increased risk. The right panel shows results for the minor allele with a protective effect. The dashed red line indicated a phenotype frequency of 1%. The barplots highlight the number of cases per phenotype in the EMR collections.
