# Supplementary material for: TYK2 Protein-Coding Variants Protect against Rheumatoid Arthritis and Autoimmunity, with No Evidence of Major Pleiotropic Effects on Non-Autoimmune Complex Traits
Source: PLoS One. 2015 Apr 7;10(4):e0122271. doi: 10.1371/journal.pone.0122271 (PMC4388675; doi:10.1371/journal.pone.0122271)
Supplement: S1 Table — (PDF) [file pone.0122271.s007.pdf]

S1 Table. Description of the subjects included in this study.

Phenotype	Dataset	Description	Cases	Controls
RA	Immunochip	Sample size	7,222	15,870
		Gender, Female %	73	57
		RF/CCP,sero-positive %	100	*NA*
	Exomechip	Sample size	4,726	13,683
		Gender, Female %	56	57
	Exon sequencing	Sample size	1,118	1,118
		Gender, Female %	77	62
		RF/CCP,sero-positive %	73.5	*NA*
SLE	Exomechip	Sample size	2,319	13,687
		Gender, Female %	90	57
IBD	Exomechip	Sample size	1,346	13,687
		Gender, Female %	57	57
EMR binary and quantitative traits (PheWAS)	i2b2, Immunochip	Sample size	1,203 *	1,802
	BioVU, Exomechip	Sample size	1,032 *	25,340

RF, rheumatoid factor ; CCP, cyclic citrullinated peptide ; RA, rheumatoid arthritis ; SLE, systemic lupus erythematosus ; IBD, inflammatory bowel disease ; EMR, electronic medical records ; PheWAS, phenome-wide association study

* *Subjects with PheWAS code 714.1 = Rheumatoid arthritis*
