# Supplementary material for: TYK2 Protein-Coding Variants Protect against Rheumatoid Arthritis and Autoimmunity, with No Evidence of Major Pleiotropic Effects on Non-Autoimmune Complex Traits
Source: PLoS One. 2015 Apr 7;10(4):e0122271. doi: 10.1371/journal.pone.0122271 (PMC4388675; doi:10.1371/journal.pone.0122271)
Supplement: S2 Table — (PDF) [file pone.0122271.s008.pdf]

S2 Table. Meta-analysis of Immunochip, Exomechip and Sequencing RA association results.

	Immunochip [a]		Exomechip [b]		Sequencing [c]		Meta-analysis	
SNP	OR (95% CI)	P	OR (95% CI)	P	OR (95% CI)	P	OR (95% CI)	P
rs34536443	0.62 (0.55-0.70)	2.2e-14	0.70 (0.61-0.79)	6.0e-8	0.68 (0.47-0.99)	0.044	0.66 (0.60-0.72)	2.3e-21
rs35018800 [d]	0.42 (0.31-0.56)	4.0e-9	0.69 (0.51-0.93)	0.016	0.52 (0.21-1.32)	0.17	0.53 (0.43-0.65)	1.2e-9
rs12720356 [e]	0.87 (0.81-0.94)	5.2e-4	0.87 (0.80-0.96)	0.003	0.73 (0.58-0.93)	0.0099	0.86 (0.82-0.91)	4.6e-7

[a] *7,222 ACPA+ RA cases and 15,870 controls of European ancestry ; results from meta-analysis of 6 collections*
[b] *4,726 RA cases and 13,683 controls of European ancestry*
[c] *1,118 RA cases and 1,118 controls of European ancestry*
[d] *Association results conditional on rs34536443*
[e] *Association results conditional on rs34536443 and rs35018800*
