# Supplementary material for: TYK2 Protein-Coding Variants Protect against Rheumatoid Arthritis and Autoimmunity, with No Evidence of Major Pleiotropic Effects on Non-Autoimmune Complex Traits
Source: PLoS One. 2015 Apr 7;10(4):e0122271. doi: 10.1371/journal.pone.0122271 (PMC4388675; doi:10.1371/journal.pone.0122271)
Supplement: S3 Table — (PDF) [file pone.0122271.s009.pdf]

S3 Table. Detailed description of the samples including in the sequencing study.

Collection	RA cases	Controls
AMC/VUMC/Reade (The Netherlands)	70	174
ABCoN (USA)	28	56
BRAGGSS (UK)	70	88
DREAM/NBS (The Netherlands)	184	186
CORRONA (USA)	87	-
i2b2 (USA)	-	159
LUMC (The Netherlands)	43	25
Biobanco-IMM **(**Portugal)	356	249
ReAct (France)	280	181
**TOTAL**	**1,118**	**1,118**
