# Supplementary material for: TYK2 Protein-Coding Variants Protect against Rheumatoid Arthritis and Autoimmunity, with No Evidence of Major Pleiotropic Effects on Non-Autoimmune Complex Traits
Source: PLoS One. 2015 Apr 7;10(4):e0122271. doi: 10.1371/journal.pone.0122271 (PMC4388675; doi:10.1371/journal.pone.0122271)
Supplement: S4 Table — (PDF) [file pone.0122271.s010.pdf]

S4 Table. Gene-based association results, restricted to nonsense and missense variants with MAF<0.5% and predicted to be possibly or probably damaging in PolyPhen-2 and SIFT.

Gene	N variants	Burden		FRQWGT		VT		SKAT-O
		direction	P	direction	P	direction	P	P
CDC37	3	risk	0.5	Protect	0.5	Protect	0.69	1
ICAM1	9	risk	0.4	Protect	0.36	Protect	0.1	0.18
ICAM3	11	risk	0.5	risk	0.46	risk	0.49	1
ICAM4	2	Protect	**0.015**	Protect	**0.015**	Protect	**0.015**	0.06
ICAM5	3	Protect	0.31	Protect	0.31	Protect	0.31	1
PDE4A	10	Protect	0.12	Protect	0.20	Protect	0.17	0.24
RAVER1	14	Protect	0.41	Protect	0.5	risk	0.39	0.46
S1PR5	6	risk	0.31	risk	0.37	risk	0.5	0.91
TYK2	22	Protect	0.12	Protect	0.15	Protect	0.18	0.38
