# Supplementary material for: TYK2 Protein-Coding Variants Protect against Rheumatoid Arthritis and Autoimmunity, with No Evidence of Major Pleiotropic Effects on Non-Autoimmune Complex Traits
Source: PLoS One. 2015 Apr 7;10(4):e0122271. doi: 10.1371/journal.pone.0122271 (PMC4388675; doi:10.1371/journal.pone.0122271)
Supplement: S5 Table — (PDF) [file pone.0122271.s011.pdf]

S5 Table. Description of the 26 missense variants predicted to be damaging identified in *TYK2* by sequencing of 1,118 RA cases and 1,118 controls.

			Counts					Protein
**CHR**	**hg19**	**rsID**	**cases**	**controls**	**Ref**	**Alt**	**Substitution**	**domain**
**chr19**	**10488926**	**rs55762744**	**14**	**27**	C	T	**A53T**	**FERM**
chr19	10488904	.	1	0	A	G	I60T	FERM
chr19	10478825	.	0	1	C	T	R124H	FERM
chr19	10477229	.	0	1	C	T	A165T	FERM
chr19	10475595	.	1	0	G	A	R381W	FERM
chr19	10475333	.	0	1	G	A	R442W	-
chr19	10473232	.	1	0	C	T	R490H	SH2
chr19	10472988	.	0	1	C	T	G541R	-
chr19	10472760	.	1	0	G	C	I589M	Kinase1
chr19	10472598	rs140594440	1	0	C	T	V603M	Kinase1
**chr19**	**10469975**	**rs12720356**	**133**	**174**	A	C	**I684S**	**Kinase1**
chr19	10469919	rs55882956	1	3	G	A	R703W	Kinase1
chr19	10468777	.	0	1	C	T	R738Q	Kinase1
chr19	10468759	.	0	1	C	T	R744Q	Kinase1
chr19	10468732	.	0	1	G	A	P753L	Kinase1
chr19	10468708	.	1	2	C	A	G761V	Kinase1
chr19	10468526	.	0	1	C	T	A794T	Kinase1
chr19	10468465	rs143743593	0	1	G	A	P814L	Kinase1
chr19	10467283	.	1	0	G	A	R860C	Kinase1
chr19	10464892	.	0	1	T	C	S912G	Kinase2
**chr19**	**10464843**	**rs35018800**	**7**	**13**	G	A	**A928V**	**Kinase2**
chr19	10464730	.	1	0	A	C	C966G	Kinase2
chr19	10463121	.	1	0	T	C	S1103G	Kinase2
**chr19**	**10463118**	**rs34536443**	**48**	**70**	G	C	**P1104A**	**Kinase2**
chr19	10461517	.	1	0	A	C	V1186G	-
chr19	10461514	.	0	1	C	T	C1187Y	-
