# Supplementary material for: TYK2 Protein-Coding Variants Protect against Rheumatoid Arthritis and Autoimmunity, with No Evidence of Major Pleiotropic Effects on Non-Autoimmune Complex Traits
Source: PLoS One. 2015 Apr 7;10(4):e0122271. doi: 10.1371/journal.pone.0122271 (PMC4388675; doi:10.1371/journal.pone.0122271)
Supplement: S6 Table — (PDF) [file pone.0122271.s012.pdf]

**S6 Table. Domain-based association results, restricted to missense variants in the protein kinase 1 domain-coding region of *TYK2* with MAF<0.5% and predicted to be possibly or probably damaging.**

| Test | N variants | P |
|---|---|---|
| Burden | 7 | 0.010 |
| VT | 7 | 0.012 |
| FqWT | 7 | 0.006 |
| SKAT-O | 7 | 0.018 |
