# Supplementary material for: TYK2 Protein-Coding Variants Protect against Rheumatoid Arthritis and Autoimmunity, with No Evidence of Major Pleiotropic Effects on Non-Autoimmune Complex Traits
Source: PLoS One. 2015 Apr 7;10(4):e0122271. doi: 10.1371/journal.pone.0122271 (PMC4388675; doi:10.1371/journal.pone.0122271)
Supplement: S10 Table — (PDF) [file pone.0122271.s016.pdf]

S9 Table. Results of association between RA-protecting *TYK2* variants and two quantitative traits: low-density lipoproteins levels and white blood cell counts.

		BioVU				i2b2 [1]				Meta-analysis	
SNP	AA change	N	BETA	(95%CI)	P	N	BETA	(95%CI)	P	BETA	P
**A. LDL [a]**											
rs34536443	P1104A	9637	3.14	(0.57-5.71)	0.017	2211	5.17	(-1.34-11.68)	0.12	3.41	**0.0051**
rs35018800	A928V	9639	-1.99	(-7.60-3.62)	0.49	2215	-2.54	(-22.07-16.99)	0.80	-2.03	0.46
rs12720356	I684S	9638	-1.13	(-2.93-0.66)	0.22	2212	-2.25	(-6.10-1.60)	0.25	-1.33	0.11
**B. WBC [b]**											
rs34536443	P1104A	25563	0.12	(-0.021-0.26)	0.095	3097	0.10	(-0.27-0.48)	0.59	0.12	0.079
rs35018800	A928V	25581	-0.27	(-0.59-0.06)	0.11	3101	0.18	(-0.94-1.29)	0.76	-0.23	0.15
rs12720356	I684S	25580	0.046	(-0.056-0.15)	0.38	3098	-0.097	(-0.32-0.12)	0.39	0.0094	0.88

LDL, low-density lipoprotein ; WBC, white blood cell counts ; AA, amino acid ; CI, confidence interval ; N, number of individuals included in the analysis ; BETA, effect size relative to the minor (RA-protective) allele

[a] *First LDL measure (mg/dL), adjusted by age at measurement, year of measurement, gender, principal components ; restricted to individuals who were not recorded as being under prescription for an HMG-CoA reductase inhibitor (statin) prior to measurement*

[b] *Mean white blood cell counts (1000 cells/mL) , adjusted by age, gender, number of measurements, principal components*

[1] *RA status was also included as a covariate in the analyses of the i2b2 collection*
